# Path-planning with waiting in spatiotemporally-varying threat fields

**DOI:** 10.1371/journal.pone.0202145

**Published:** 2018-08-23

**Authors:** Benjamin S. Cooper, Raghvendra V. Cowlagi

**Affiliations:** Aerospace Engineering Program, Worcester Polytechnic Institute, Worcester, MA, United States of America; Nanjing University of Information Science and Technology, CHINA

## Abstract

We address the problem of finding an optimal path for a vehicle in a planar environment where traversal costs are based on a time-varying spatial field defined over the environment. The resulting optimal path may contain instances of *waiting*, where the vehicle hovers, parks, or loiters. First, we consider path-planning on a uniform grid over the workspace. It is known that the computational complexity of the problem is significantly higher when waiting is allowed. We study the trade-off between the increased computational complexity and potential cost reductions in the resultant path with allowance for waiting. The results of numerical studies in this work identify characteristics of the threat fields in which optimal paths can involve waiting. Furthermore, we provide a local condition on the threat field that *precludes* waiting from providing any cost reductions in the resultant path. We show that this condition can be used in the path-planning algorithm to prune search trees and provide significant reductions in computation time without significant suboptimality. Next, we consider path-planning on a vehicle-centric multiresolution grid. We use a wavelet-based multiresolution decomposition to evaluate the multiresolution path planner and compare against the uniform resolution grid using the same family of threat fields. We show that with a vehicle-centric multiresolution map and an appropriate path-planning algorithm, the added computational effort of allowance for waiting is negligible.

## 1 Introduction

Consider the problem of planning a driving route from one place to another. Minimum-time solutions to this problem are provided by many commercially available gadgets [1] and freely available software applications such as Google Maps [2]. Furthermore, such applications are now capable of using real-time traffic data to provide estimates of the travel duration [3]. Table 1 shows estimated travel durations between the cities Springfield, MA, Boston, MA, and an intermediate town Millbury, MA, which is approximately equidistant from Springfield and Boston. The city halls of each city are chosen as specific starting and ending street addresses. These data are obtained using the Google Maps application on a desktop computer browser, queried at 06:15 AM and again at 07:07 AM on April 10, 2018. Notice the following peculiarity of this result. Per the 06:15 AM query, the estimated travel duration from Springfield to Millbury, MA is 52 min, and that from Millbury to Boston is 1 hr. 11 min. A vehicle starting from Springfield at 06:15 AM will ostensibly reach Millbury at 07:07 AM. However, in the 07:07 AM query, the estimated travel duration from Millbury to Boston increases by 18%, and the total travel duration from Springfield to Boston will be larger than the 06:15 AM estimate of 2 hrs. 1 min. An explanation of this discrepancy is that the Google Maps application possibly ignores temporal variations in traffic. The algorithmic details of the Google Maps application are proprietary and not publicly known.

**Table 1:** Estimated travel times obtained from the Google Maps application. Data obtained on April 10, 2018.

	Spr. to Bos.	Spr. to Mil.	Mil. to Bos.
Query at 06:15 AM	2 hrs. 3 min.	52 min	1 hr. 11 min.
Query at 07:07 AM	2 hrs. 16 min.*	–	1 hr. 24 min.

*: This duration is calculated by adding the Spr. → Mil. duration to the Mil.→ Bos. duration estimated in the 07:07 AM query.

Consider now that the objective of the same path planning problem were not to minimize travel duration, but rather to minimize a weighted sum of travel duration and exposure to traffic. Such an objective may be of importance to reduce the health risks to long-haul truck drivers by reducing their exposure to automobile emissions [4–6]. This problem is also of renewed importance because in recent times, a wealth of traffic data has become available to make accurate predictions of traffic [7, 8]. In this case, an optimal route may involve *waiting* (e.g. at a rest area) for traffic to subside.

This problem is an example of the general problem of path-planning problem with minimum exposure to a scalar field (e.g. traffic density in this example) that varies with space and time. Other applications of this problem include motion-planning for aerial vehicles in inclement weather with physics-based predictive models, and for mobile robots with continually updated estimates of workspace features from noisy measurements.

**Fig 1:**
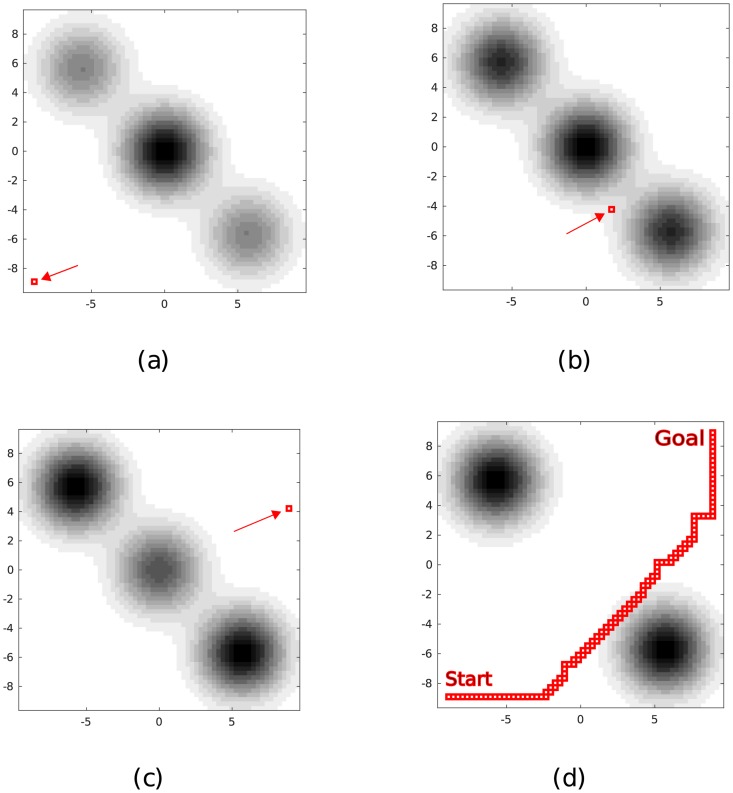
An illustration of the abstract path-planning problem studied in this article. Snapshots of the threat field at different time instants are shown, where higher threat intensities are indicated by darker colors. The vehicle’s location as it moves through the field is indicated by a red dot. (a) Snapshot at *t* = 0 s. (b) Snapshot at *t* = 50 s. (c) Snapshot at *t* = 100 s. (d) Snapshot at *t* = 115 s. The path shown in (d) is a *non-waiting* minimum exposure path, which will be discussed in the sequel.

To serve these motivating applications, we investigate the abstract problem of path-planning *including waiting for finite intervals of time*, in a 2D workspace in the presence of a spatiotemporal threat field, where minimal exposure to the threat is desired, as illustrated in Fig 1. The term “threat” is a generic term to refer to any scalar quantity of interest. The canonical path- and motion-planning problems in the existing literature are of finding obstacle-free paths and/or trajectories between prespecified initial and destination points [9]. Geometric methods [10, 11], randomized sampling-based methods [12, 13], trajectory optimization methods [14], and combinations thereof [15, 16] are reported in the literature to solve these problems. Problems involving predictive models of changes in the environment, where the planner can actively choose to *wait* for a more “favorable” environment are less thoroughly studied. The robotics literature addresses path-planning with workspace cost maps [17], but the extension of this approach to time-varying cost maps is challenging.

We consider first a scenario where the threat field is fully known, and then a scenario where the field is partially known in vehicle-centric multiresolution detail. The solutions to these problems are conceptually simple: the dimension of the search space is expanded to include the temporal dimension. However, computational implementations are challenging because the search space along this temporal dimension is in principle unbounded even if the 2D spatial workspace is compact.

### 1.1 Related work

A standard practice to path-planning is the application of Dijkstra’s algorithm or the A* algorithm in a discretized 2D workspace [9, 18], and the point of departure in different approaches is the manner of discretization. Examples of such discretization include quadtree-like workspace cell decompositions [10, 11], visibility roadmaps [19], and other multiresolution grids [20, 21]. In this context, path-planning in a spatiotemporal threat field is formulated by assigning appropriate time-varying edge costs in the associated topological graph.

Optimal path-finding algorithms for graphs with time-varying edge costs appear in [22–25]. The applications reported in the literature of such algorithms are primarily for transportation [26, 27], and for data transmission in networks [24, 28, 29]. The typical objective in such applications is minimum time or minimum delay [30].

An example in which an optimal path can involve waiting is of a passenger at a railway station who must decide whether to board a slower train available immediately or to wait for an express train scheduled for later [30]. An important result is that waiting is never beneficial for minimum time path-planning if the so-called First-In-First-Out (FIFO) condition is satisfied [31]: namely, that departing a position later will not result in arriving earlier (the FIFO condition may not hold for the express train example). However, this condition is in general not satisfied if the cost is different from travel duration, and therefore optimal paths can in general involve waiting. General algorithms for path-planning with waiting to minimize time-varying edge costs appear in [22, 31, 32].

In this paper, we discuss path-planning to minimize a cost defined by the sum of movement cost, waiting cost, and exposure to a spatiotemporal threat. This threat is assumed to be known over the spatial domain of interest (i.e. the vehicle’s workspace) and at all time instants. First, we consider path-planning on a uniform grid over the workspace. It is known that the computational complexity of the problem is higher when waiting is considered [31]. We study the trade-off between the increased computational complexity and potential cost reductions in the resultant path by considering waiting. The results of numerical studies in this work identify characteristics of the field for which optimal paths can involve waiting. Furthermore, we provide a local condition on the threat field that *precludes* waiting from providing any cost reductions in the resultant path. Next, we consider path-planning on a vehicle-centric multiresolution grid. Recent efforts to utilize non-uniform grids to find optimal paths in spatiotemporal environments include selectively ignoring the time dimension [33] and adaptively discretizing the search grid with respect to variations in a velocity field [34]. However, such multiresolution planning schemes work best when large regions are static [33] or do not explicitly evaluate waiting [34]. We use a wavelet-based multiresolution decomposition to evaluate the multiresolution path planner and compare against the uniform-resolution case using the same family of threat fields.

### 1.2 Contributions

The contributions of this paper are towards improving the computational efficiency of path-planning algorithms for mobile vehicle applications with time-varying changes in the vehicle’s environment. First, we evaluate the benefit of waiting in a family of threat fields constructed as linear combinations of Gaussian basis functions. Because all square integrable functions can be approximated with arbitrary precision by such Gaussian linear combinations, the results of this paper can be used to train machine learning algorithms to determine for a given application *a priori* whether or not an optimal path will involve waiting. Second, we present a condition based on local threat field characteristics, to be incorporated in graph search algorithms, that allows pruning search trees that cannot result in an optimal path. We comment on the application of this condition towards path cost reduction and with respect to computational expense through numerical studies. Third, we study the influence of a vehicle-centric *multiresolution* threat field map, and demonstrate that the added computational expense of considering waiting is negligible if waiting is allowed only in the immediate spatial vicinity of the vehicle. To the best of the authors’ knowledge, there is no literature on multiresolution path-planning with allowance for waiting.

Preliminary results of this work were reported in [35], which addressed a special case of general problem of path-planning in a time-varying spatial field addressed in this paper. Specifically, [35] did not address waiting, which is the primary focus of this paper. Furthermore, results for only for one representative case study were reported in [35], whereas this paper evaluates the solution over *several thousand* different randomly generated environments. Therefore, this paper significant advances over [35].

The rest of this paper is organized as follows. Mathematical formulations of the aforesaid problem of interest are presented in Section 2 and its solution methods, including the local no-wait condition, are presented in Section 3. The main results are presented in 4, with conclusions and comments on future work in Section 5.

## 2 Problem formulations

In what follows, $R^{}$ and $Z^{}$ represent the sets of reals and integers, respectively.

### 2.1 Threat field parametrization

We consider a threat field constructed as the weighted sum of a finite number of 2D Gaussian basis functions $\bar{c}\left(x,t\right)=\sum_{n=1}^{N_{P}}w_{n}\left(t\right)\phi_{n}\left(x,t\right).$ Here, the spatial basis function *ϕ*_*n*_ is defined for each *n* = 1, …, *N*_P_ by

$$\begin{array}{c} \phi_{n}(x,t)≔\frac{1}{\sqrt{2\pi }}\exp(\frac{-{\left(x-\mu_{n}\left(t\right)\right)}^{T}\left(x-\mu_{n}\left(t\right)\right)}{2∥\Sigma_{n}{\left(t\right)∥}^{2}}), \end{array}$$ (1)

where *μ*_*n*_(*t*) = (*μ*_*nx*_(*t*), *μ*_*ny*_(*t*)) defines the spatial mean of *ϕ*_*n*_ and Σ_*n*_(*t*) = (*σ*_*nx*_(*t*), *σ*_*ny*_(*t*)) defines the spatial spread of *ϕ*_*n*_. In this work, we consider affine functions *μ*_*n*_ and Σ_*n*_ of the form *μ*_*n*_(*t*) = *μ*_*n*0_ + *μ*_*n*1_*t*, and Σ_*n*_(*t*) = Σ_*n*0_ + Σ_*n*1_*t*, where $\mu_{n0},\mu_{n1},\Sigma_{n0},\Sigma_{n1}\in R^{2}$ are prespecified constants.

The finite parameterization of the threat field $\bar{c}$ using Gaussian functions is justified by the fact that a large class of functions on $R^{}$, namely, square integrable functions, can be approximated with arbitrary precision by linear combinations of Gaussian functions [36]. Furthermore, 2D Gaussian functions of varying width can be used to represent varying levels of resolution by including narrower Gaussian functions into higher resolution images or video [37]. Finally, Gaussian function appear in series solutions to several partial differential equations such as the diffusion equation [38], which enables the application of the proposed work to path- and motion-planning with threat field modeled by physical phenomena such as advection-diffusion of gases or radiation in the atmosphere [39].

### 2.2 Path-planning with uniformly high resolution field map

Let $W\subset R^{2}$ be a closed square region, called the *workspace*, in which the vehicle moves. We consider a strictly positive spatiotemporal scalar field $\bar{c}:W\times [0,\infty )\to R_{>0}^{},$ called the *threat field*, which represents unfavorable regions with higher intensity. For path-planning, we restrict time to a compact interval $T=[t_{0},t_{f}]\subset R_{+}^{}$. The threat field may represent, for instance, terrain elevation [40], a risk measure [21], a probabilistic occupancy grid [41], or the atmospheric concentration of a gas [39].

First, we consider path-planning on a grid consisting of $N_{G}^{2}$ points uniformly placed in *N*_G_ rows and *N*_G_ columns. The coordinates in a prespecified Cartesian coordinate axis system of the *j*^th^ grid point are denoted by x^*j*^, for each $j=1,...,N_{G}^{2}$. The vehicle is assumed to traverse grid points according to a “4–connectivity” rule, and the time taken to traverse between adjacent grid points is a prespecified constant *t*_step_. In this paper, we neglect vehicle kinematic and dynamic constraints that can restrict this motion, while noting that such constraints can in the future be incorporated in the proposed grid-world problem setup [16].

We define a graph $\bar{G}=\left(\bar{V},\bar{E}\right),$ where each vertex in $\bar{V}$ is uniquely associated with a grid point, and labeled with superscripts as ${\bar{v}}^{1},{\bar{v}}^{2},\ldots ,{\bar{v}}^{N_{G}^{2}}.$ The edge set $\bar{E}$ is defined as the set of pairs of vertices associated with adjacent grid points. A *path between vertices* ${\bar{v}}^{i_{s}},{\bar{v}}^{i_{g}}\in \bar{V}$, denoted $\bar{v}\left(i_{s},i_{g}\right),$ is a finite sequence of vertices $\left({\bar{v}}_{0},{\bar{v}}_{1},\ldots ,{\bar{v}}_{P}\right)$, with ${\bar{v}}_{0}={\bar{v}}^{i_{s}},$ ${\bar{v}}_{P}={\bar{v}}^{i_{g}},$ and either $\left({\bar{v}}_{j-1},{\bar{v}}_{j}\right)\in \bar{E}$ or ${\bar{v}}_{j-1}={\bar{v}}_{j},$ for each *i* ∈ {1, …, *P*}. Note that *sub*scripts are used to denote indices of vertices. The *cost* of the path $\bar{v}\left(i_{s},i_{g}\right)$ is defined by $\bar{J}(\bar{v})≔\sum_{j=1}^{P}\bar{g}\left(\left({\bar{v}}_{j-1},{\bar{v}}_{j}\right),jt_{\text{step}}\right),$ where $\bar{g}:\bar{E}\times [0,\infty )\to R_{>0}^{}$ is a strictly positive function that assigns time-varying edge transition costs. Specifically,

$$\begin{array}{c} \bar{g}\left(\left({\bar{v}}^{k},{\bar{v}}^{\ell }\right),t\right)=\{\begin{array}{cc} \bar{c}\left(x^{\ell },t\right)+\alpha_{m}, & \;\text{if}\;{\bar{v}}^{k}\neq {\bar{v}}^{\ell },\\ \bar{c}\left(x^{\ell },t\right)+\alpha_{w}, & \;\text{if}\;{\bar{v}}^{k}={\bar{v}}^{\ell }. \end{array} \end{array}$$ (2)

where *α*_m_, and *α*_w_ are prespecified strictly positive constants. Note that the preceding definition of a path and its cost implicitly allows for *waiting* at any grid point, namely, instances where ${\bar{v}}_{j-1}={\bar{v}}_{j}$ for any *j* ∈ {1, …, *P*}. The constants *α*_w_ and *α*_m_ are costs of waiting and of moving, respectively.

**Problem 1.** *Find a path* ${\bar{v}}^{*}\left(i_{s},i_{g}\right)$ *such that* $\bar{J}({\bar{v}}^{*}\left(i_{s},i_{g}\right))\leq \bar{J}(\bar{v}\left(i_{s},i_{g}\right))$ *for every other path* $\bar{v}\left(i_{s},i_{g}\right)$ *in* $\bar{G}.$

### 2.3 Path-planning with vehicle-centric multiresolution field map

**Fig 2:**
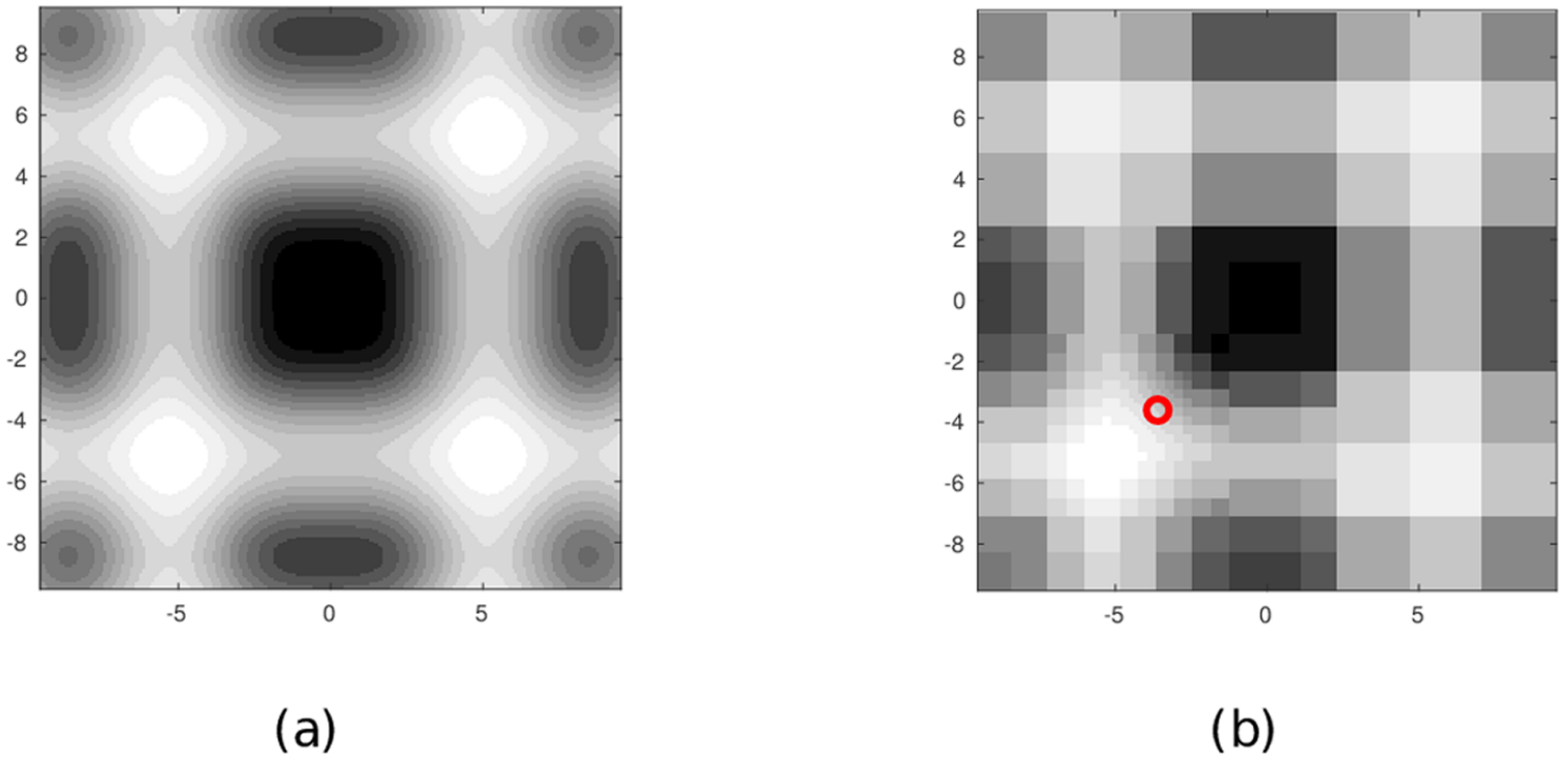
Example of an intensity map and its vehicle-centric multiresolution approximation according to Eq (8). The vehicle’s location is indicated by the black dot near the center. (a) Original field map. (b) Vehicle-centric multiresolution approximation.

We consider next a multiresolution discretization of the workspace based on the discrete wavelet transform (DWT). This multiresolution discretization algorithm is adopted from the second author’s previous works [35, 42], and provides a so-called *vehicle-centric multiresolution approximation* to the threat field intensity map. The motivation for considering multiresolution discretization is twofold. First, such a discretization may be necessary for practical onboard computations. Second, such a discretization reflects the nature of typical maps available to vehicles in many applications: namely, well-known in the immediate vicinity of the vehicle, partially known in regions farther away, as illustrated in Fig 2. We investigate the potential cost reductions by considering waiting in path-planning. Waiting is allowed only at vertices in the highest resolution region described in Section 2.3. We provide a skeletal overview of this discretization here, and refer the reader to [42] for details.

The DWT represents a scalar field using so-called *approximation and detail coefficients*, which multiply spatial basis functions called *scaling functions and wavelets*. If appropriate, it is possible to use these spatial basis functions to also represent the threat field, without affecting the results presented in this paper. The reasons for using wavelets in this section are the convenient dyadic structure and orthogonality of the basis functions [43].

Without loss of generality, we assume that W=[0,1]×[0,1]. For the following discussion, we choose a parameter $D\in Z_{+}^{}$ indicating the highest resolution considered in the multiresolution approximation. Specifically, the smallest grid separation (i.e. highest resolution) is 2^−*D*^. For context, the grid separation in the uniformly high resolution map considered in the previous subsection is 2^−7^ units.

**Assumption 1.** The field $\bar{c}$ is sufficiently smooth such that

$$\begin{array}{c} \bar{c}\left(x,y,t_{0}\right)+\sum_{n=1}^{\infty }\frac{{\left(t-t_{0}\right)}^{n}}{n!}\frac{\partial^{n}\bar{c}\left(x,y,t\right)}{\partial t^{n}}{|}_{t=t_{0}} \end{array}$$ (3)

converges to $\bar{c}\left(x,t\right)$ for all x∈W, and *t* ∈ [0, ∞).

**Assumption 2.** The values taken by $\bar{c}$ are known at a finite resolution *m*_f_ > −*D*, for $k,\ell =0,1,\ldots ,2^{D+m_{f}}-1$. Without loss of generality, *m*_f_ = 0.

**Assumption 3.** The values taken by the temporal derivatives of $\bar{c}$ at time *t* = *t*_0_ are known at the same finite resolution as described in Assumption 2.

Relying on Assumption 1, we consider Taylor series expansions of *time-varying* approximation and detail coefficients of the DWT of the threat field $\bar{c}.$ The coefficients of these Taylor series expansions are given by

$$\alpha_{m_{0},k,l}^{n}≔\langle \Phi_{m_{0},k,l}\left(x,y\right),\frac{\partial^{n}\bar{c}\left(x,y,t\right)}{\partial t^{n}}{|}_{t=t_{0}}\rangle ,$$ (4)

$$\beta_{m,k,l}^{p,n}≔\langle \Psi_{m,k,l}^{p}\left(x,y\right),\frac{\partial^{n}\bar{c}\left(x,y,t\right)}{\partial t^{n}}{|}_{t=t_{0}}\rangle ,$$ (5)

for *p* = 1, 2, 3, $k,\ell \in Z^{},$ *m*_f_ ≥ *m* ≥ *m*_0_ = −*D*, and $n\in Z_{⩾0}^{}.$ Here Φ and Ψ denote families of scaling functions and wavelets, respectively (see [42] for details). The threat field is reconstructed from these coefficients as follows:

$$\begin{array}{cc} \hat{c}\left(x,y,t\right) & =\sum_{k,\ell =0}^{1}\sum_{n\in Z_{⩾0}^{}}\frac{{\left(t-t_{0}\right)}^{n}}{n!}\alpha_{m_{0},k,\ell }^{n}\Phi_{m_{0},k,\ell }\left(x,y\right)\\ & +\sum_{p=1}^{3}\sum_{m=m_{0}}^{m_{f}}\sum_{k,\ell =0}^{2^{m-m_{0}}}\sum_{n\in Z_{⩾0}^{}}\frac{{\left(t-t_{0}\right)}^{n}}{n!}\beta_{m,k,\ell }^{p,n}\Psi_{m,k,\ell }^{p}\left(x,y\right), \end{array}$$ (6)

where $\hat{c}$ denotes the reconstructed field. To construct a vehicle-centric multiresolution approximation of the threat field, let $A\subset \{\left(m,k,\ell \right)\in Z^{3}:m_{0}\leq m<0,\;0\leq k,\ell \leq 2^{D+m}\}$. We define for all *p* = 1, 2, 3 and $n\in Z_{+}^{}$,

$$\begin{array}{c} {\hat{\beta }}_{m,k,\ell }^{p,n}≔\{\begin{array}{cc} \beta_{m,k,\ell }^{p,n} & (m,k,\ell )\in A,\\ 0 & \;\text{otherwise.} \end{array} \end{array}$$ (7)

The reconstruction of the threat field is then performed using ${\hat{\beta }}_{m,k,\ell }^{p,n}$ in Eq (6) instead of $\beta_{m,k,\ell }^{p,n}.$ The set A contains the indices of detail coefficients that are considered “significant”. Following [42], we choose A such that high resolution information is retained in the immediate vicinity of the vehicle’s current location $(x_{0},y_{0})\in W$ and it is gradually discarded in regions farther away. To this end, let $\varrho :Z^{}\to N^{}$ be a “window” function that specifies, for each level of resolution, the distance from the vehicle’s location up to which the detail coefficients at that level are significant. The set $A=A^{\text{win}}\left(x_{0},y_{0}\right)$ of indices is then defined by

Awin(x0,y0)≔{dm,k,ℓp(m,k,ℓ):m0≤m<0,⌊2mx0⌋-ϱ(m)≤k≤⌊2mx0⌋+ϱ(m),⌊2my0⌋-ϱ(m)≤ℓ≤⌊2my0⌋+ϱ(m)dm,k,ℓp}. (8)

The preceding description of the vehicle-centric multiresolution approximation is minimal; the interested reader is referred to [35, 42] for further details. In what follows, we focus on the following problem.

**Problem 2.** *Solve Problem 1 using a vehicle-centric multiresolution approximation* $\hat{c}$ *of the threat field*.

To formulate Problem 2 precisely, we define a multiresolution cell decomposition Ω^mr^, which is a partition of W into square cells of different sizes, such that $\hat{c}$ is constant in the spatial variables over each of the cells. We denote by $\text{cell}({\bar{v}}^{k};\Omega )$ the coordinates of the center of the cell associated with a vertex ${\bar{v}}^{k}\in \bar{V}$, and by $\text{vert}(C;\bar{G})$ the vertex in $\bar{V}$ associated with a cell C∈Ω. We attach with the cell decomposition Ω^mr^ a graph G=(V,E) such that each cell in Ω^mr^ corresponds to a unique vertex in *V*. Each vertex *j* ∈ *V* corresponds to a set $W\left(v^{k},V\right)\subset \bar{V}$, and the collection {*W*(*v*^*k*^, *V*)}_*v*^*k*^∈*V*_ is a partition of $\bar{V}$. Specifically:

$$\begin{array}{c} W(v^{k},V)≔\{{\bar{v}}^{k}\in \bar{V}:\text{cell}\left({\bar{v}}^{k};\Omega \right)\subseteq \text{cell}\left(v^{k};\Omega^{\text{mr}}\right)\}. \end{array}$$ (9)

Vertices *v*^*k*^, *v*^ℓ^ ∈ *V* are adjacent in G if there exist ${\bar{v}}^{k}\in W\left(v^{k},V\right)$ and ${\bar{v}}^{\ell }\in W\left(v^{\ell },V\right)$ such that $\left\{{\bar{v}}^{k},{\bar{v}}^{\ell }\right\}\in \bar{E}$. We define edge costs $g:E\to R_{+}^{}$ in G as

$$\begin{array}{cc} g(\left(v^{k},v^{\ell }\right),t) & ≔\{\begin{array}{cc} \bar{c}\left(x^{\ell },t\right)+\alpha_{w}, & \text{if}\;|W\left(v^{k},V\right)|=1,v^{k}=v^{\ell },\\ \left(c_{v^{\ell }}\left(v^{\ell },t\right)+\alpha_{m}\right)\left|W\left(v^{\ell },V\right)\right|, & \text{otherwise,} \end{array} \end{array}$$ (10)

where *c*_*v*^ℓ^_ are time-averaged cell intensities. Owing to the use of the DWT, the computation of these time-averaged intensities involves simple algebraic computations (see [35] for details). This edge cost function considers approximate periods of time and distance required to traverse these cells, which are in turn related to the cell sizes |*W*(*v*^ℓ^, *V*)|. We restrict waiting to only the uniform resolution window of Ω^mr^, precisely, when |*W*(*v*^*k*^, *V*)| = 1 and *v*^*k*^ = *v*^ℓ^ in Eq (10). The cost J(v) of a path in G is the sum of all edge costs.

## 3 Methods

### 3.1 General solution to Problem 1

Problem 1 is similar to a standard path-planning problem with the exception that the edge transition costs are time-varying. This exception, however, significantly complicates the solution of this problem. Specifically, to solve Problem 1, a search must be performed not on the graph $\bar{G}$ but instead on a much larger graph $\bar{G}T$ obtained as follows. Let T be a line graph whose vertices are the time instants *t*_0_, *t*_1_, …, and whose edges are the pairs (*t*_*j*−1_, *t*_*j*_), where *t*_*j*_ − *t*_*j*−1_ = *t*_step_ for each $j\in N^{}.$ The graph $\bar{G}T$ is then defined as the *product* of graphs $\bar{G}$ and T, i.e., each vertex in $\bar{G}T$ is a pair $({\bar{v}}^{k},t_{j}),$ and the pairs $\left({\bar{v}}^{k},t_{j}\right)$ and $\left({\bar{v}}^{\ell },t_{m}\right)$ form an edge of $\bar{G}T$ if and only *t*_*m*_ − *t*_*j*_ = *t*_step_ and either ${\bar{v}}^{k}={\bar{v}}^{\ell }$ or $\left({\bar{v}}^{k},{\bar{v}}^{\ell }\right)\in \bar{E},$ with $k,\ell \in \{1,\ldots ,N_{G}^{2}\}$ and $j,m\in N^{}.$ The worst-case complexity of Dijkstra’s algorithm for solving Problem 1 is $O(|\bar{G}T|(1+\log|\bar{G}T|)).$

In typical path-planning algorithms, Dijkstra’s algorithm is sped up using a *search heuristic*: for example, the A* algorithm uses Euclidean distance to the goal as a search heuristic [18]. For Problem 1, we can define for each vertex ${\bar{v}}^{k}\in \bar{V},$ $k=1,2,\ldots ,|\bar{V}|,$ we define the search heuristic

$$\begin{array}{c} h({\bar{v}}^{k})≔\alpha_{m}∥x^{i_{g}}-x^{k}∥, \end{array}$$ (11)

which is Euclidean distance to the goal scaled by the positive constant *α*_m_. The scale factor ensures that the heuristic *h* underestimates the true cost to the goal, and therefore ensures that the search algorithm returns an optimal path [18]. Note that the *worst-case* computational complexity of the search algorithm does not change despite using the search heuristic [18], although a significant speed-up is achieved for most $\left({\bar{v}}^{i_{s}},{\bar{v}}^{i_{g}}\right)$ pairs of initial and goal vertices.

### 3.2 No-wait path-planning

An approximate solution to Problem 1 can be obtained by ignoring the possibility of waiting. To this end, we define a *no-wait path* as a path $\bar{v}=\left({\bar{v}}_{0},{\bar{v}}_{1},\ldots ,{\bar{v}}_{P}\right)$ where ${\bar{v}}_{j-1}\neq {\bar{v}}_{j}$ for each *i* ∈ {1, …, *P*}. We define an edge transition cost function:

$$\begin{array}{c} {\bar{g}}_{\text{nw}}(\left({\bar{v}}^{k},{\bar{v}}^{\ell }\right),t)≔\bar{c}\left(x^{\ell },t\right)+\alpha_{m}. \end{array}$$ (12)

The cost of a no-wait path is then defined as ${\bar{J}}_{\text{nw}}(\bar{v})≔\sum_{j=1}^{P}{\bar{g}}_{\text{nw}}\left(\left({\bar{v}}_{j-1},{\bar{v}}_{j}\right),jt_{\text{step}}\right).$

Compared to solving Problem 1 *exactly* as in Section 3.1, it is significantly easier to find a path ${\bar{v}}_{\text{nw}}^{*}\left(i_{s},i_{g}\right)$ with minimum no-wait cost ${\bar{J}}_{\text{nw}}\left({\bar{v}}_{\text{nw}}^{*}\right).$ The worst-case complexity of Dijkstra’s algorithm to find this path is $O(|\bar{G}\left|(1+\log\right|\bar{G}|)),$ and $|\bar{G}|$ is smaller than $|\bar{G}T|$ by the order of magnitude of |T|.

It is therefore natural to question: (1) whether the computational effort in *exactly* solving Problem 1 is worthwhile: i.e., whether the suboptimality of the ${\bar{v}}_{\text{nw}}^{*},$ measured as $\bar{J}({\bar{v}}_{\text{nw}}^{*})-\bar{J}({\bar{v}}^{*}),$ is large enough to justify spending the significantly higher computational resources to find the true solution ${\bar{v}}^{*}$ to Problem 1, and (2) whether the exact solution ${\bar{v}}^{*}$ can be computed faster. In the sequel we address both of these questions.

To address the first question (on computational effort), we characterize the difference $\bar{J}({\bar{v}}_{\text{nw}}^{*})-\bar{J}({\bar{v}}^{*})$ based on a study of a large number of numerical simulations (discussed in Section 4). The results of this study identify the threat fields where the difference $\bar{J}({\bar{v}}_{\text{nw}}^{*})-\bar{J}({\bar{v}}^{*})$ is significant. This study consisted of 2000 simulations of different instances of Problem 1. In each simulation, the threat field $\bar{c}$ was constructed with an arbitrary *N*_*P*_ chosen from the set {3, 4, …, 40}, followed by arbitrary choices of the constants *w*_*n*0_, *w*_*n*1_, *μ*_*n*0_, *μ*_*n*1_, Σ_*n*0_, and Σ_*n*1_ for each *n* = 1, …, *N*_P_. The weights *w*_*n*0_, *w*_*n*1_ were fixed at 1 so that all peaks in the field are of uniform height. The parameters *μ*_*n*0_, *μ*_*n*1_ were chosen by random sampling on a uniform distribution over the workspace W. The parameters Σ_*n*0_, Σ_*n*1_ were chosen by random sampling from a uniform distribution a quarter the size of the workspace W. The simulations were run using $N_{G}^{2}=1600$, *t* = [0, 100], and $t_{\text{step}}=\frac{W_{\text{wd}}}{N_{G}-1}$ where $W_{\text{wd}}$ is the width of the (square) workspace. The product graph $\bar{G}T$ in each simulation has approximately 1.2 × 10^6^ vertices. The simulations were designed using MATLAB^®^ and executed on a Windows 7 Enterprise^®^ computer using an Intel^®^ Core^*TM*^ i7-4770 3.4 GHz CPU and 16 GB RAM. The results of this study are discussed in Section 4, and MATLAB^®^ source code is available at https://github.com/rvcowlagi/waiting.

The issue of interest here is whether it is beneficial to search in the larger product graph $\bar{G}T$ or the smaller topological graph G. This issue depends on the properties of the threat field itself, and not on the specific software implementation of the search algorithm. Therefore, simulation results in MATLAB^®^ suffice to study this issue. Real-time implementations of the search algorithms (e.g. in a lower-level language such as C/C++) are not necessary because they shed no further light.

To address the second question (whether the exact solution can be computed faster), we develop a method to prune search trees during the execution of Dijkstra’s algorithm as discussed next.

### 3.3 Local test for no-wait suboptimality

The number of computations in solving Problem 1 can be reduced by pruning edges of $\bar{G}T$ during the execution of Dijkstra’s algorithm. Specifically, an edge of $\bar{G}T$ between the pairs $\left({\bar{v}}^{k},t_{j}\right)$ and $\left({\bar{v}}^{k},t_{j+1}\right)$, $k\in \left\{1,\ldots ,N_{G}^{2}\right\},j\in N^{},$ can be pruned if a condition, as identified next, precludes waiting at x^*k*^ at time *t*_*j*−1_ from being optimal.

**Fig 3:**
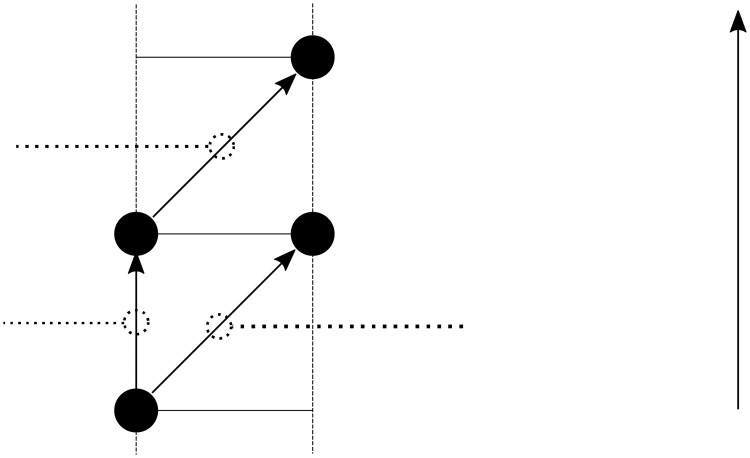
Illustration of the local no-wait condition. The decision to wait at vertex *v*^*k*^ or immediately move to vertex *v*^ℓ^ is based on the future threat field values $\bar{c}\left(x^{k},t_{j+1}\right)$, $\bar{c}\left(x^{\ell },t_{j+1}\right)$, $\bar{c}\left(x^{\ell },t_{j+2}\right)$, and waiting and movement costs *α*_w_ and *α*_m_.

Consider the cost of moving from x^*k*^ to an adjacent point x^*ℓ*^, with $k,\ell \in \{1,\ldots ,N_{G}^{2}\}.$ Accordingly, consider the vertex $\left({\bar{v}}^{k},t_{j}\right)$ of $\bar{G}T,$ and two paths for traveling to x^*ℓ*^. The first path is the single edge in $\bar{G}T$ from vertex $\left({\bar{v}}^{k},t_{j}\right)$ to vertex $\left({\bar{v}}^{\ell },t_{j+1}\right)$ achieves this travel. The second path consists of two successive edges from vertex $\left({\bar{v}}^{k},t_{j}\right)$ to vertex $\left({\bar{v}}^{k},t_{j+1}\right)$ to vertex $\left({\bar{v}}^{\ell },t_{j+2}\right)$ also achieves this travel, but includes waiting at the point x^*k*^ for one time step (see Fig 3). Waiting at x^*k*^ is beneficial if the cost of the first path is greater than the cost of the second path, i.e., if

$$\begin{array}{c} \bar{g}\left(\left({\bar{v}}^{k},{\bar{v}}^{k}\right),t_{j+1}\right)+\bar{g}\left(\left({\bar{v}}^{k},{\bar{v}}^{\ell }\right),t_{j+2}\right)<\bar{g}\left(\left({\bar{v}}^{k},{\bar{v}}^{\ell }\right),t_{j+1}\right), \end{array}$$ (13)

$$\begin{array}{c} \Rightarrow \bar{c}\left(x^{k},t_{j+1}\right)+\alpha_{w}+\bar{c}\left(x^{\ell },t_{j+2}\right)<\bar{c}\left(x^{\ell },t_{j+1}\right), \end{array}$$ (14)

For further insight into this condition, consider a two vertex system with current position x^*k*^, and goal position x^*ℓ*^. Fig 3 illustrates the possible paths and costs. For brevity, define $F_{k,j+1}≔\bar{c}\left(x^{k},t_{j+1}\right)$, $F_{\ell ,j+1}≔\bar{c}\left(x^{\ell },t_{j+1}\right)$, and $F_{\ell ,j+2}≔\bar{c}\left(x^{\ell },t_{j+2}\right).$ Waiting is beneficial if

$$\begin{array}{cc} \alpha_{w}+F_{k,j+1}+\alpha_{m}+F_{\ell ,j+2} & <\alpha_{m}+F_{\ell ,j+1}\\ \Rightarrow \alpha_{w}+F_{k,j+1} & <F_{\ell ,j+1}-F_{\ell ,j+2}, \end{array}$$ (15)

and it is easy to see that,

$$\begin{array}{c} \alpha_{w}+\bar{c}\left(x^{k},t_{j+1}\right)<-\Delta \bar{c}\left(x^{k}+\Delta x,t_{j+1}\right)\Delta t \end{array}$$ (16)

The terms in (16) suggest that an optimal path can involve waiting when the sum of *α*_w_ and the threat in the next time step $\bar{c}\left(x^{k},t_{j+1}\right)$ is less than the difference between the field costs at the next vertex $-\Delta \bar{c}\left(x^{k}+\Delta x,t_{j+1}\right)\Delta t,$ where x^*ℓ*^ = x^*k*^ + Δx. This occurs when the waiting costs and field values are low relative to large decreasing changes in the field threat. In other words, it is beneficial to wait if the threat along the optimal path ${\bar{v}}^{*}$ is rapidly diminishing and the cost to remain at current position x^*k*^ is low. This observation is supported via numerical experiments. We identify a local no-wait condition as follows.

$$\begin{array}{c} \alpha_{w}+\bar{c}\left(x^{k},t_{j+1}\right)+\bar{c}\left(x^{\ell },t_{j+2}\right)-\bar{c}\left(x^{\ell },t_{j+1}\right)\geq 0 \end{array}$$ (17)

When executing Dijkstra’s algorithm, edges can be pruned using (17). If (17) holds true, then the two-vertex local condition is violated. To decrease the number of edges explored in the graph $\bar{G}T$, the neighbors of ${\bar{v}}^{k}$ are evaluated under (17) at each iteration. If any neighbor violates the waiting condition, then ${\bar{v}}^{k}$ is marked as a non-waiting node.

### 3.4 Solution to Problem 2

The multiresolution path-planning algorithm utilizes Dijkstra’s algorithm to search the product GT of graph G and T.

**Fig 4:**
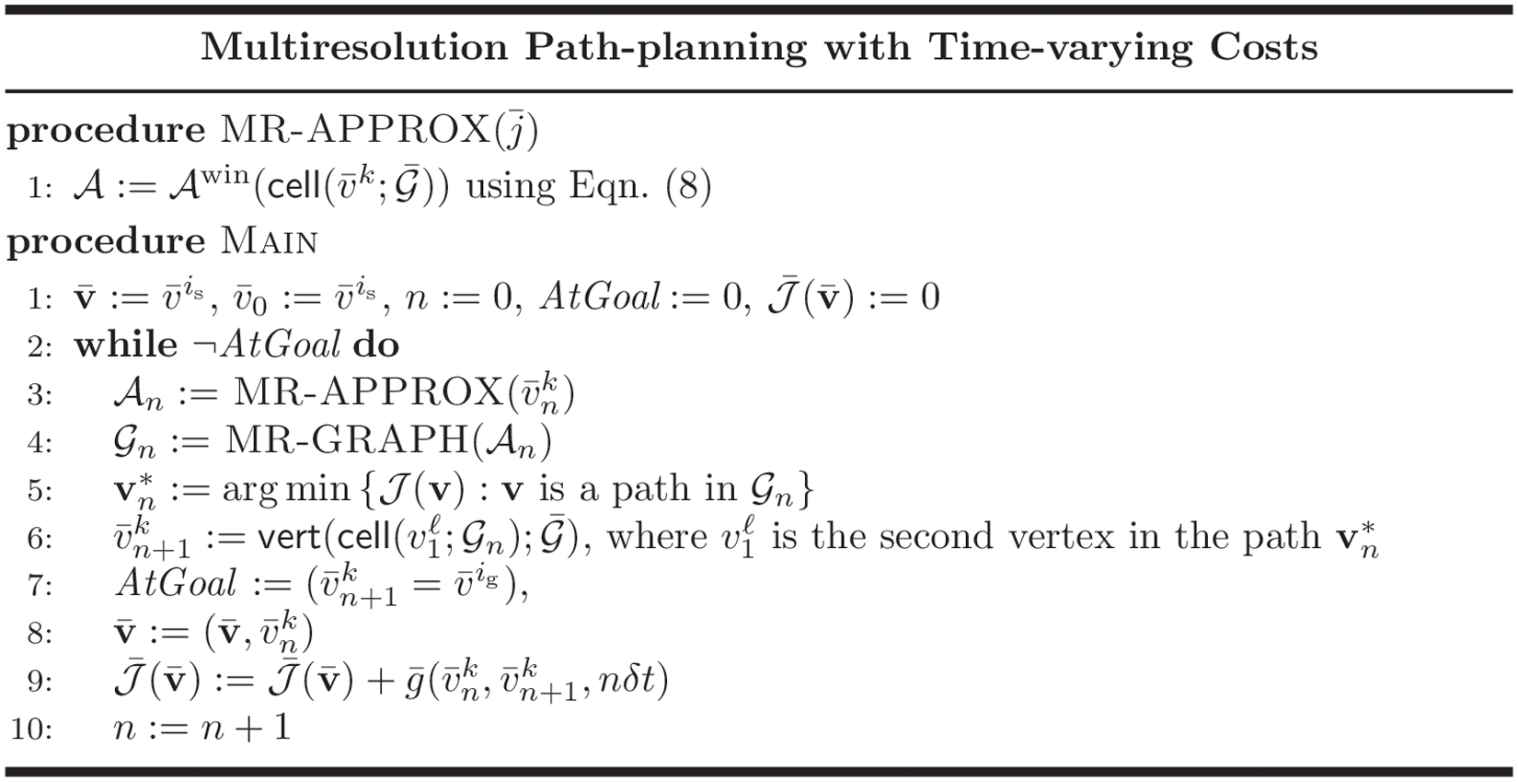
Pseudo-code for the proposed path-planning algorithm.

Fig 4 shows in pseudo-code form the proposed path-planning algorithm based on the vehicle-centric multiresolution approximation of the spatial field $\bar{c}$. The algorithm iterates Lines 3–10 until the goal is reached. At each iteration, the algorithm computes a vehicle-centric multiresolution approximation and the corresponding cell decomposition graph (Lines 1–4). In Line 5, the optimal path in $G_{n}$ is computed by a label-correcting algorithm. The vehicle is assumed to traverse the first cell in the path $\pi_{n}^{*}$, and the process is repeated for the new vehicle location.

A procedure to determine the locations and the sizes of cells in Ω^mr^ in the vehicle-centric multiresolution approximation, including fast updates of these cell locations and sizes with the changing vehicle location, is provided in [42]. Furthermore, a procedure denoted MR-GRAPH to determine the edges in the graph G associated with Ω^mr^, including fast updates to the sets of vertices and edges of this graph with the changing vehicle location, is also provided in [42].

One detail not explicitly stated in the pseudo-code in Fig 4 is the ability to bypass the optimization problem in Line 5 when the multiresolution decomposition in Lines 1–4 is unchanged. Because of the vehicle-centric decomposition, if the vehicle has found a waiting beneficial path it will remain stationary for several iterations while it steps through the optimal space-time path, v*. In this case, Line 5 can be bypassed until the vehicle moves and the decomposition updates. For the multiresolution planning, this significantly speeds up the planning and allows the planner which considers waiting to compute in the nearly the same time as the no-wait planner as will be seen in the results section.

The *topological properties* (i.e. cell locations, sizes, and adjacency relations) of the vehicle-centric multiresolution approximation are the same as in [42] and therefore, the proof of completeness of this path-planning algorithm is the same as that provided in detail in [42].

## 4 Results and discussion

The results in this section are summarized as follows. The allowance for waiting in the path-planning algorithm can reduce paths costs by 25% compared to no-wait path-planning, but this cost reduction incurs an added computational expense of a 30- to 300-fold increase in execution time. Using the local no-wait condition of Section 3.3 reduces this added computation expense to only a 10-fold increase in execution time, while discovering a majority of optimal paths. In comparison to the uniform resolution case, multiresolution discretization has strong computational benefits: specifically, the added computational expense of the allowance for waiting in path-planning is negligible.

### 4.1 Empirical study with uniformly high resolution map

In what follows, we present the results of the numerical study described in Section 3.2, specifically, the characteristics of the difference in costs between the path solving Problem 1 and the no-wait path: $\bar{J}({\bar{v}}_{\text{nw}}^{*})-\bar{J}({\bar{v}}^{*})$ normalized by $\bar{J}({\bar{v}}^{*}).$

#### Computational effort for allowance of waiting

**Fig 5:**
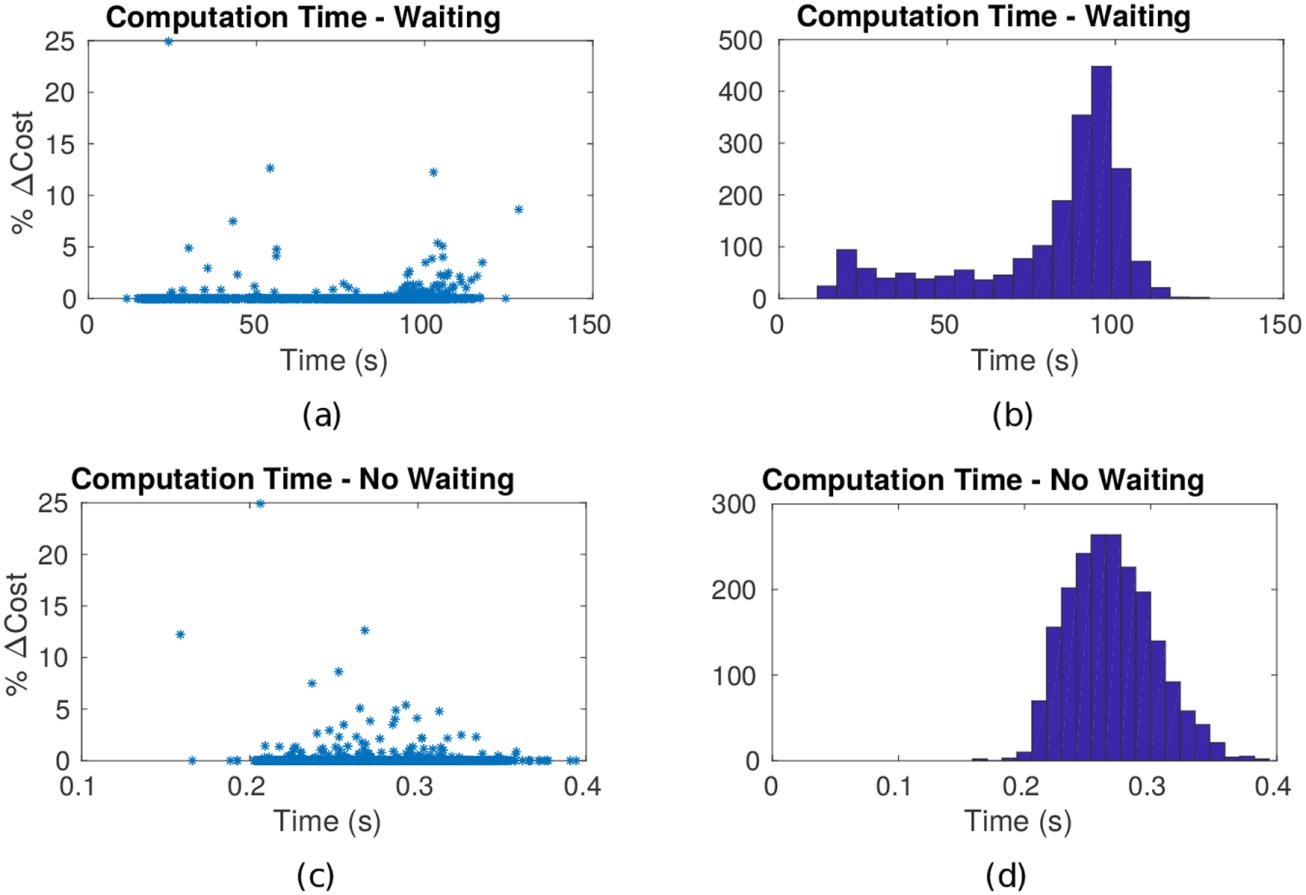
Execution time to solve Problem 1. (a),(b) The computational expense with waiting included is bimodal with the dominant mode at higher computation times. Bimodality indicates calculation of waiting paths falls into two populations: optimal waiting paths found in a narrow search of $\bar{G}T$, and optimal waiting paths found in a broader search of $\bar{G}T$. This observation identifies two characteristically different threat field families. (c),(d) Computational expense (execution time) when waiting is not allowed.

Over the 2000 simulations conducted, the greatest suboptimality of the no-wait path was 25%. In 156 of these simulations, i.e., nearly 8% of the simulated cases, ${\bar{v}}_{\text{nw}}^{*}$ was suboptimal compared to ${\bar{v}}^{*}$, but this suboptimality was significant (greater than 5%) in only in less than 1% of cases of the 2000 simulations. The computation times for finding the no-wait path in each simulation were all under 0.4 s, while those for finding the solution to Problem 1 were in the range of 11–120 s as shown in the histogram in Fig 5. In other words, the computation time to find the solution ${\bar{v}}^{*}$ to Problem 1 was between 30–300 times slower than the time to find the no-wait approximate solution ${\bar{v}}_{\text{nw}}^{*},$ whereas the suboptimality $\bar{J}({\bar{v}}_{\text{nw}}^{*})-\bar{J}({\bar{v}}^{*})$ was greater than zero in slightly less than 8% of the cases simulated. In certain applications, however, this extra computational effort may be justified, say in the transportation of hazardous waste through adverse weather (threat) [25].

#### Local no-wait condition—Cost reduction and computational efficiency

**Fig 6:**
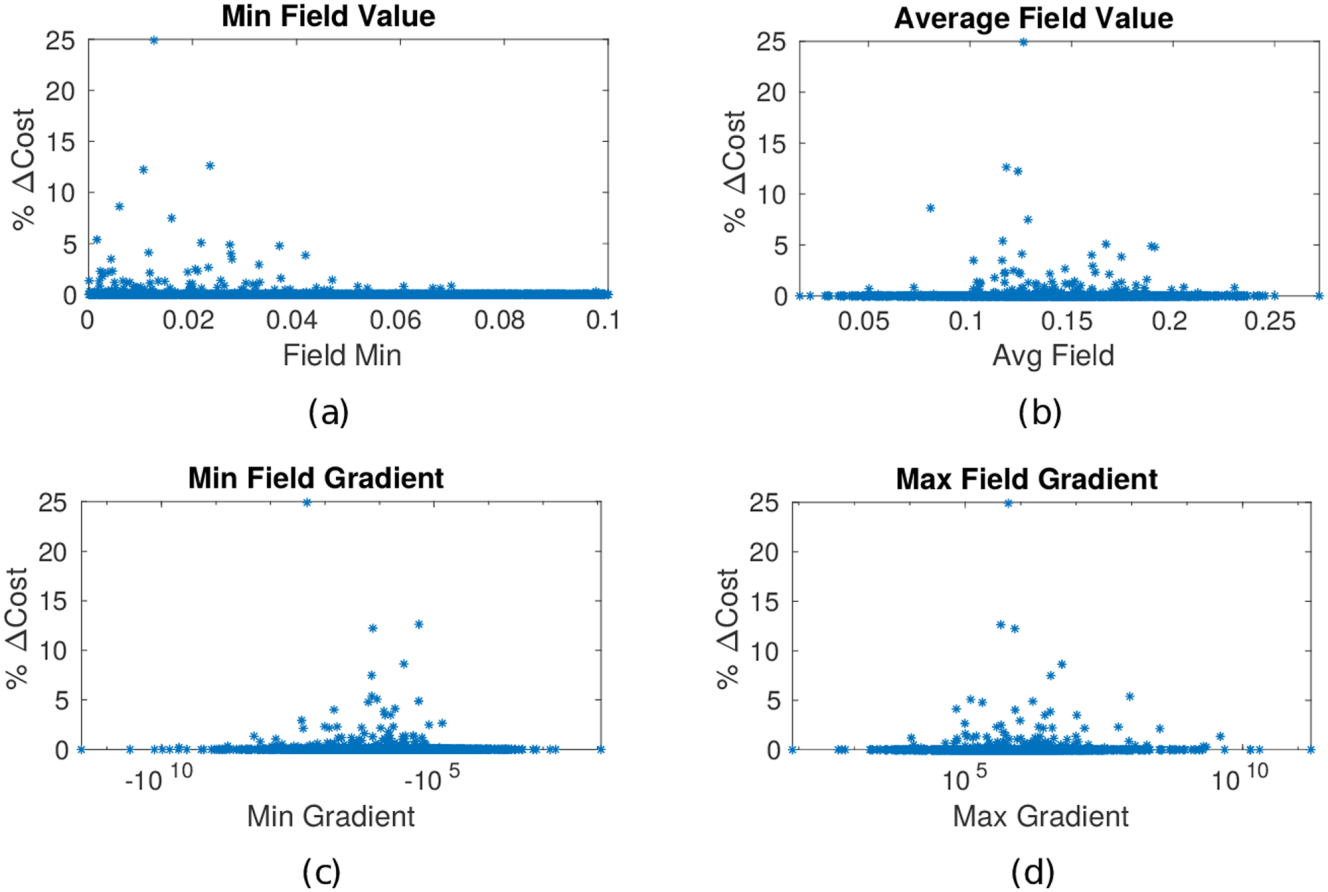
Difference in costs of waiting-allowed and no-wait optimal paths, for different field characteristics. When the minimum field value is low, optimal paths more frequently include waiting. The greatest differences between paths with and without waiting cluster near a median value suggesting that large and small temporal gradients of the field favor movement over waiting.

The discussion in Section 3.3 indicates that waiting is locally beneficial when the waiting cost *α*_w_ and the threat field value are low compared to the local temporal gradient of the threat field. It was noted that the movement cost *α*_m_ did not affect this local test, and therefore the ratio *α*_m_/*α*_w_ is not relevant to the waiting decision. In Fig 6(a), we note that the cost reduction of waiting occurs more frequently with a lower minimum values of the field $\bar{c}$. Fig 6(b)–6(d) summarize the average value across time for the field and its gradients.

When the field temporal gradient is small, i.e., $\nabla \bar{c}\left(x,t\right)\to 0$, the field is approximately static and an optimal path does not involve waiting. For further insight into the lack of waiting paths for fields with large temporal gradients, consider a vehicle at location x^*k*^ at time *t*_*j*_ and the threat value $\bar{c}\left(x^{\ell },t_{j}\right)$ at location x^*ℓ*^ along the optimal path ${\bar{v}}^{*}$. Due to the large gradient, the threat will move or dissipate at time *t*_*j*+*m*Δ*t*_. If the threat at position x^*ℓ*^ dissipates such that $\nabla \bar{c}\left(x^{\ell }\right)⩾\frac{dx}{dt}$, where $\frac{dx}{dt}$ is the speed of the vehicle, then the vehicle arrives after the threat is dissipated and no waiting is required. However, if the vehicle arrives at position x^*ℓ*^ before time *t*_*j*+*m*Δ*t*_, it will incur a threat exposure cost and should wait. These arguments indicate that optimal paths may involve waiting when the field changes moderately over time, i.e., $\nabla \bar{c}\left(x,t\right)$ is neither too high nor too low.

**Fig 7:**
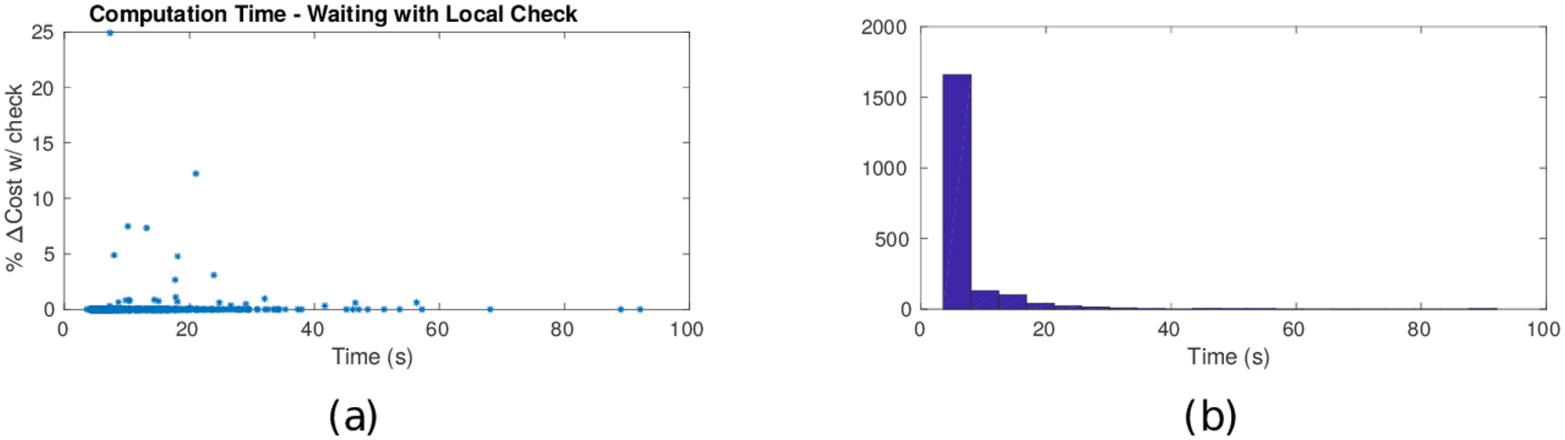
Computational efficiency due to local no-wait condition. When the local no-wait condition (17) is used to prune search trees the calculation time for the majority of paths is significantly reduced while still finding optimal paths that include waiting.

As discussed in Section 3.3, we can modify Dijkstra’s algorithm with the local no-wait condition (16), which leads to a significant computational advantage. Using this local no-wait condition check, the average calculation time is reduced from 78.7s to 5.47s, which is a 140% reduction (see Fig 7). With the local no-wait condition check, 83% of paths are determined in under 6 seconds while still discovering optimal paths that involve waiting. Because this no-wait condition is approximate, potentially optimal waiting paths can be discarded. Among the 2000 simulations conducted, 156 cases involved waiting in the optimal path, but with the local no-wait condition check included, only 13 of these 156 waiting paths were found by the algorithm. However, these included paths with the greatest reduction in cost: specifically, the reduction of cost of the waiting paths (compared to an optimal no-wait path) with a cost difference greater than 5%, 57% were found by the algorithm including the local no-wait check condition. In summary, the local no-wait condition was observed to significantly reduce computational efforts while finding optimal paths in a majority of the simulated cases.

#### Other dependencies

**Fig 8:**
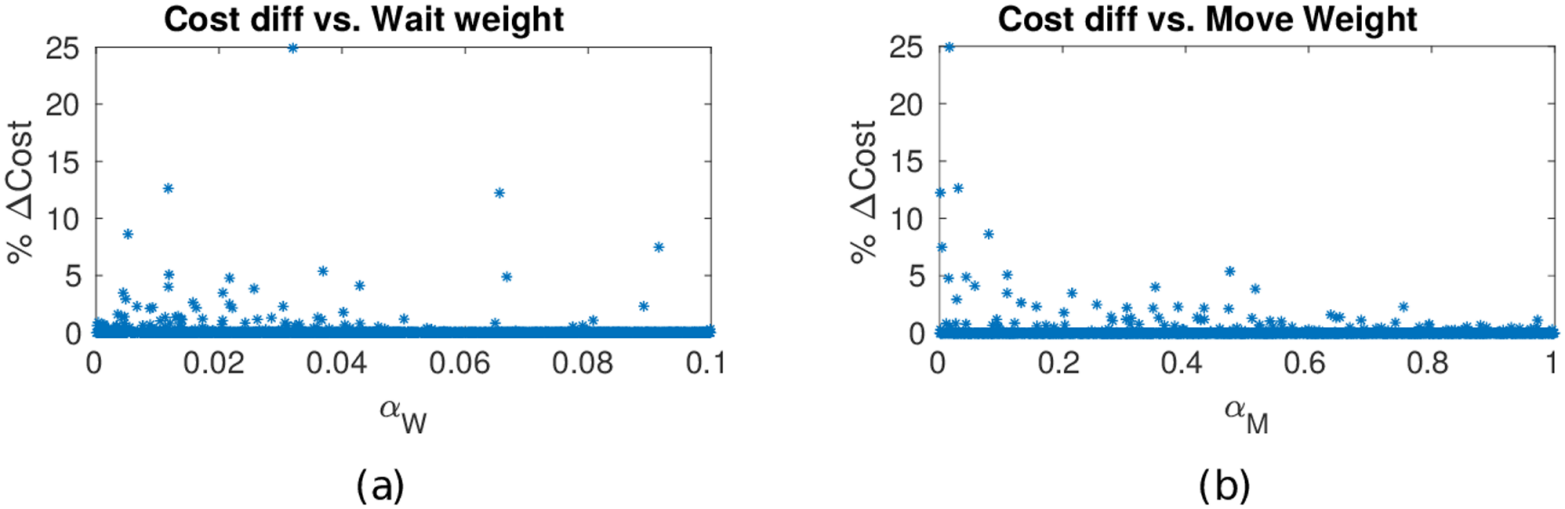
Difference in costs of waiting-allowed and no-wait optimal paths, for different values of constants in edge transition cost. For lower values of the constants *α*_w_ and *α*_m_, optimal paths more frequently involve waiting.

Fig 8 indicates that a low value of the movement cost constant *α*_m_ in the edge transition cost (2), the optimal path more frequently involves waiting. Such cases may occur when the optimal no-wait path ${\bar{v}}_{\text{nw}}^{*}$ is shorter (i.e., has fewer vertices) but has higher path cost $\bar{J}({\bar{v}}_{\text{nw}}^{*})$ compared to when that ${\bar{v}}_{w}^{*}$ is longer than ${\bar{v}}_{\text{nw}}^{*},$ but has lower cost path due to threat exposure. For these simulations the waiting cost constant *α*_w_ was restricted to the interval (0, 0.1].

**Fig 9:**
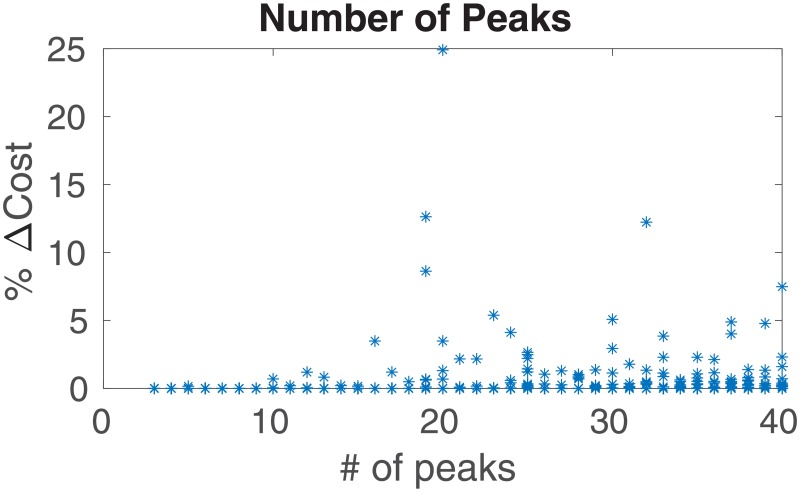
Difference in costs of waiting-allowed and no-wait optimal paths, for different numbers of field parameters. For fields with a larger number of parameters (i.e. peaks) *N*_P_, the optimal path more frequently involves waiting.

The number of parameters *N*_P_ used to define the threat field also demonstrated a pattern with respect to cost-reduced waiting paths. In Fig 9, an increase in *N*_P_ is associated with a greater number of optimal paths involving waiting. An increase in the density of peaks in the field may lead to lower spatial gradients of the field (min and max), which influences optimal waiting-allowed paths in a manner similar to the aforesaid influence of the temporal gradient.

### 4.2 Empirical study with vehicle-centric multiresolution map

**Fig 10:**
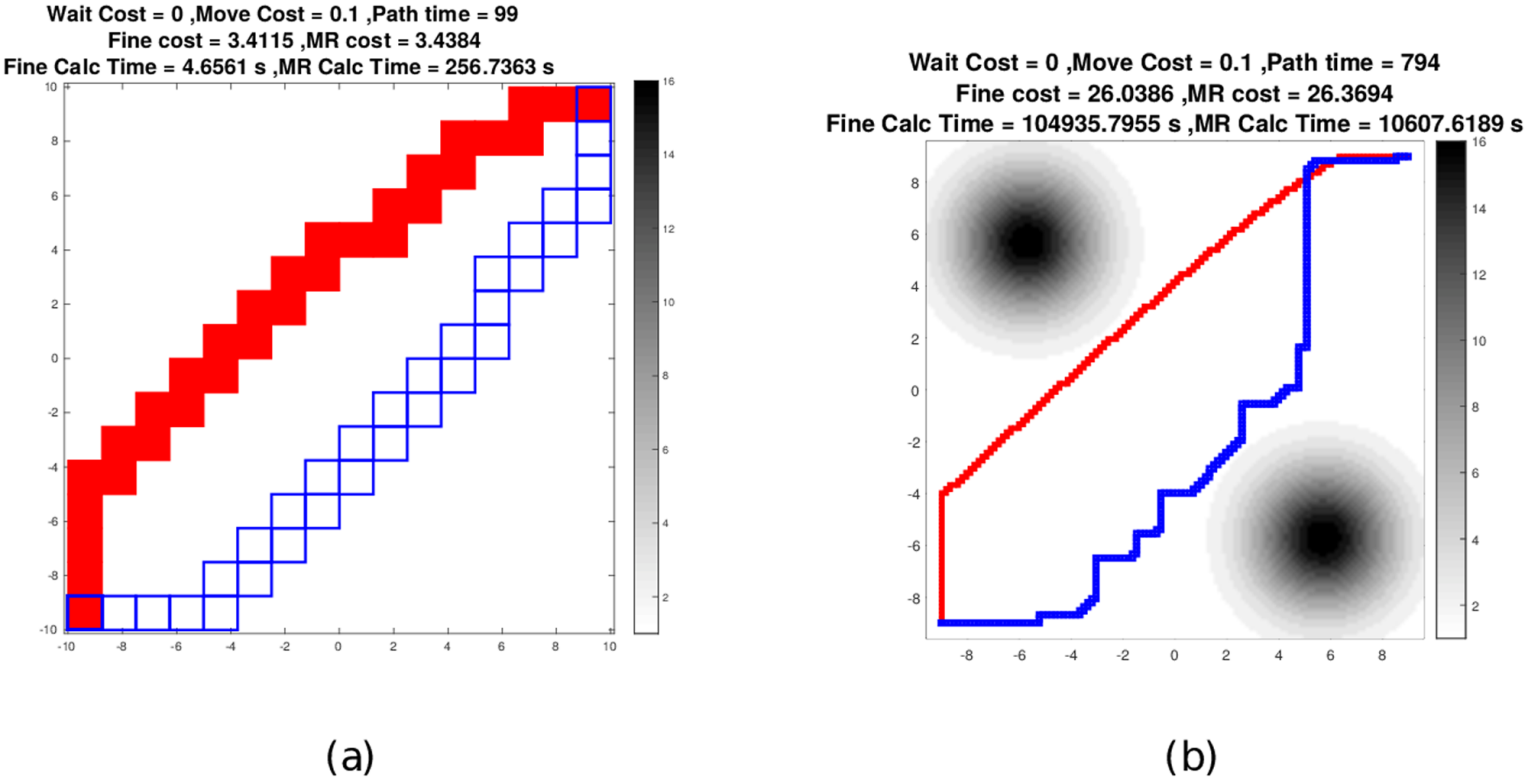
Waiting-allowed path with a multiresolution map of a three peak Gaussian field. Here, the center peak fades over time for *D* = 4 and 7 (a and b). The multiresolution path is in blue boxes and the uniform resolution path in solid red. Note the tenfold reduction in calculation time for the *D* = 7 multiresolution case.

An investigation with the multiresolution representation was initially performed using a threat field with three parameters (i.e., *N*_P_ = 3) as shown in Fig 10, corresponding to three “peaks” of the threat field. The center peak decreases in magnitude over time, therefore the optimal path should wait as long as possible then traverse through the center of the field. This scenario was run at four different resolutions, *N*_G_ = {16, 32, 64, 128}, corresponding to *D* = {4, 5, 6, 7} and the results of these simulations are summarized in Table 2. Recall that the parameter *D* indicates the highest resolution considered in the multiresolution approximation. With *D* = 6 the total computation time for finding the optimal path with the multiresolution map (i.e. solving Problem 2) is of the same order of magnitude as that for finding the optimal path in the uniform resolution map ((i.e. solving Problem 1)). With *D* = 7, computation with the multiresolution map is an order of magnitude faster. This computational speed is achieved with suboptimality, i.e. the cost of the solution of Problem 2 is always greater than or equal to the cost of solution of Problem 1. However, with *D* = 7, the maximum such suboptimality observed across all cases was approximately 1%. Next, we discuss this suboptimality with lower values of *D*.

**Table 2:** Summary of cost of and calculation times.

		Allow wait		No wait	
*D*		Uniform	Multires.	Uniform	Multires.
4	Path cost	3.41	3.43	3.57	3.88
	Calc. time	4.66 s	256.7 s	0.79 s	92.1 s
5	Path cost	6.57	6.64	7.39	7.42
	Calc. time	59.9 s	743.9 s	13.0 s	358.0 s
6	Path cost	12.91	13.04	14.72	14.72
	Calc. time	2305 s	2489 s	375.8 s	1284.2 s
7	Path cost	26.04	26.37	29.62	29.76
	Calc. time	104,940 s	10,600 s	21,835 s	5,978 s

**Fig 11:**
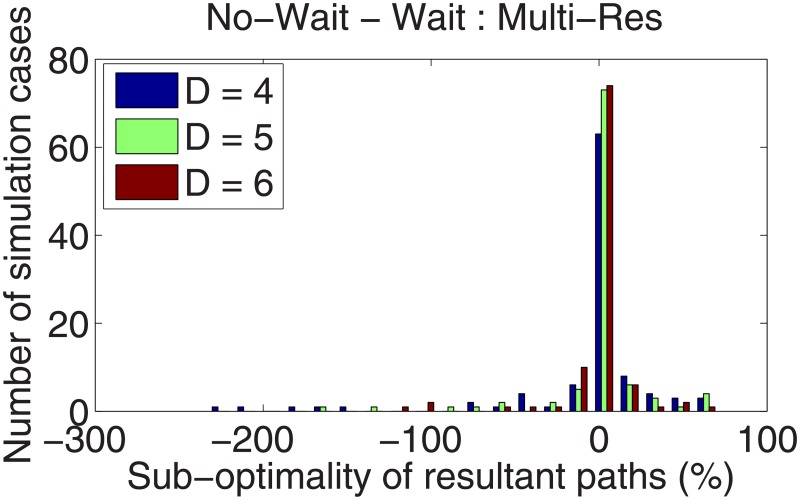
Percentage cost reduction when waiting is considered using the multiresolution representation at different resolution levels. Negative cost reductions (or sub-optimality of no-wait paths) indicate that the algorithm anticipated a benefit from waiting but was mistaken.

**Fig 12:**
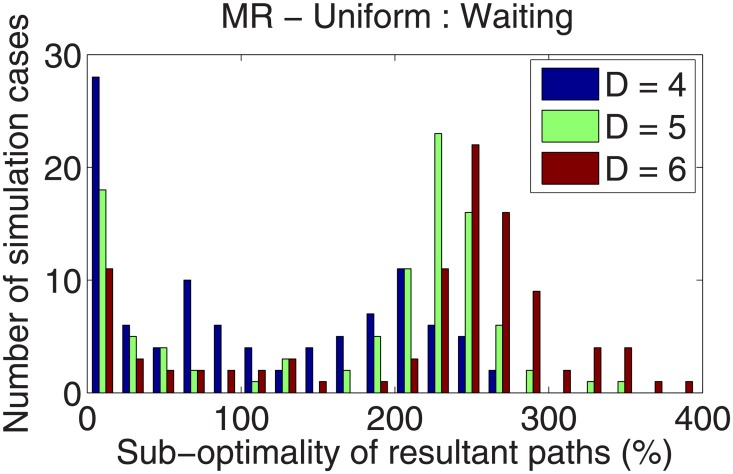
Suboptimality of using the multiresolution map, with different values of the parameter *D*.

In addition to the three peak field, the numerical study reported in the Section 4.1 was repeated for the multiresolution cases *D* = {4, 5, 6}. All parameters of the threat fields remain the same. A set of 100 simulations (in addition to the previous set of 2000 simulations) was run for each *D* = {4, 5, 6}, and each simulation included four calculations: waiting with uniform and multiresolution and non-waiting with uniform and multiresolution. The major results are summarized in Figs 11 and 12.

Fig 11 describes the cost reduction of considering a path with waiting under the multiresolution approximation of the field. A significant difference from the uniform resolution results is the phenomenon of cases when the allowance for waiting is leads to paths with *higher* costs compared to no-wait paths. An explanation of this phenomenon is as follows. Based on the current position, the path planner expects that waiting is beneficial but ends up anticipating incorrectly due to the partial knowledge available at that location. However, increasing the *D* parameter reduces the number of such occurrences.

Fig 12 shows the difference between uniform- and multiresolution cases when both path planners allow waiting, i.e., the difference in the cost of the solution of Problem 2 to that of Problem 1. Therefore, this result is an indication of the suboptimality incurred due to the vehicle-centric multiresolution approximation of the field. This suboptimality increases with increasing values of *D*. This observation is explained as follows. Allowance for waiting is made inside of the high-resolution window of the multiresolution approximation. As *D* increases, the area of this window is a smaller portion of the overall workspace, and therefore the multiresolution path planner is “myopic” and suboptimal.

**Fig 13:**
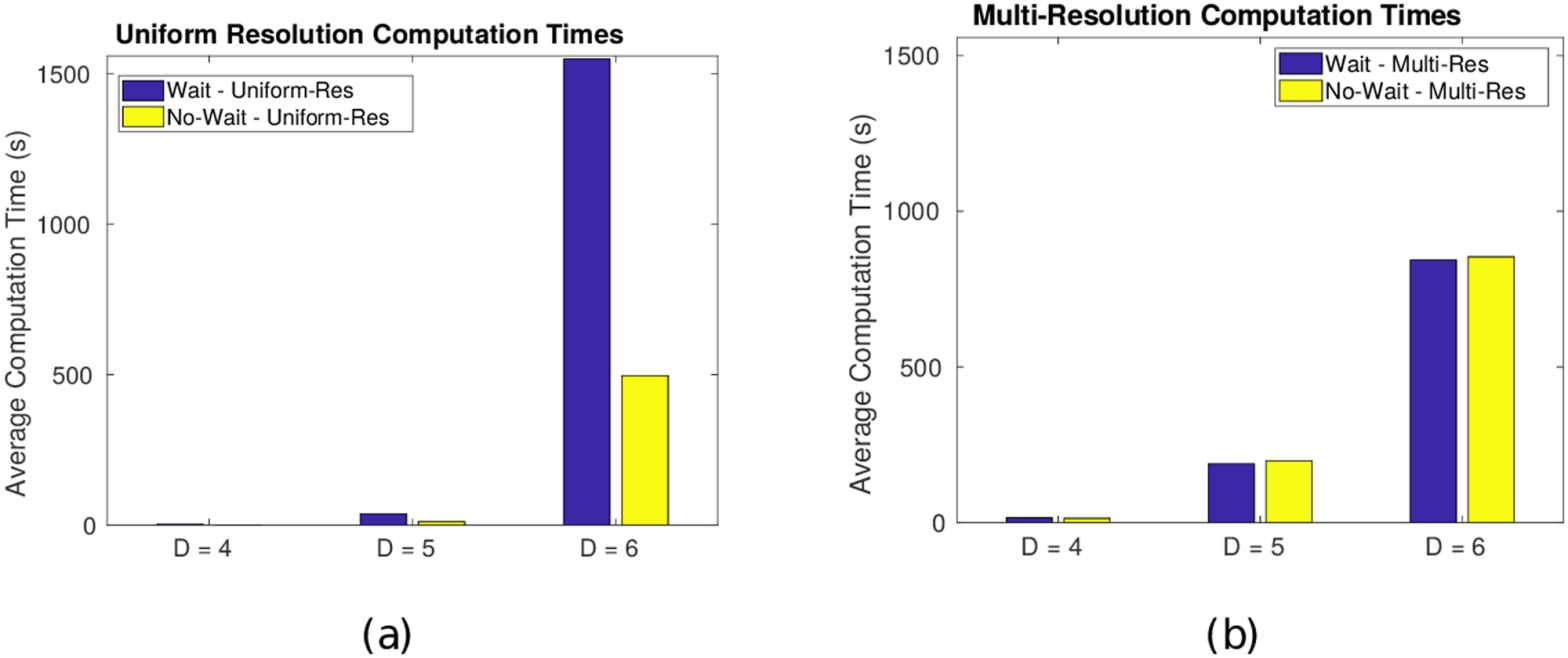
Comparison of computation times. The computation time was measured and averaged for each case of waiting-allowed vs. no-wait paths, and uniform vs. multiresolution maps. Waiting-allowed and no-wait computations with multiresolution maps require equal computational time.

Finally, we observe the average computation times for the four cases of waiting-allowed vs. no-wait paths and uniform vs. multiresolution maps, as summarized in Fig 13. It is immediately clear that the multiresolution map bears computational advantages as the resolution parameter *D* increases. Specifically, *the average computation time between waiting and non-waiting for the multiresolution cases is nearly equal*. This result was previously alluded to when describing the path planning algorithm in Fig 4. Whenever the search algorithm explores waiting, the *spatial* multiresolution decomposition does not change. Therefore, in the multiresolution path-planning problem, the allowance of waiting does not incur significant additional computational expense.

## 5 Conclusions

In this paper, we studied the inclusion of waiting at vertices in path-planning for minimizing exposure to a time-varying threat field. We proposed a local no-wait condition based on the threat field characteristics, which decides if waiting should be considered at the current vertex in the search algorithm. Through numerical simulations, the threat field characteristics when optimal paths can involve waiting were explored and the local no-wait condition was demonstrated. The local no-wait condition was successful in reducing the majority of test case’s computation time by 140% while finding an optimal path in a majority of the simulated cases. We extended the application of multiresolution path planning to allow waiting and found that the vehicle-centric multiresolution approximation of the threat still allowed the discovery of beneficial waiting paths. Importantly, we observed that including waiting in to the multiresolution planner has a negligible computational expense.
